# Aflatoxin B1 Induced Oxidative Stress and Gut Microbiota Disorder to Increase the Infection of Cyprinid Herpesvirus 2 in Gibel Carp (*Carassius auratus gibelio*)

**DOI:** 10.3390/antiox12020306

**Published:** 2023-01-28

**Authors:** Mingyang Xue, Miao Fu, Mengwei Zhang, Chen Xu, Yan Meng, Nan Jiang, Yiqun Li, Wenzhi Liu, Yuding Fan, Yong Zhou

**Affiliations:** 1 Yangtze River Fisheries Research Institute, Chinese Academy of Fishery Sciences, Wuhan 430223, China; 2 Department of Ophthalmology, Renmin Hospital of Wuhan University, Wuhan 430060, China; 3 Department of Aquatic Animal Medicine, College of Fisheries, Huazhong Agricultural University, Wuhan 430070, China

**Keywords:** aflatoxin B1, oxidative stress, intestinal microbiota, cyprinid herpesvirus 2, *Carassius auratus gibelio*, susceptibility

## Abstract

Aflatoxin contamination of food and water is a serious problem worldwide. This study investigated the defensive ability of gibel carp exposed to aflatoxin B1 (AFB1) by challenging it with cyprinid herpesvirus 2 (CyHV-2) infection. The data showed that AFB1 exposure significantly increased the mortality of CyHV-2-infected gibel carp, and enhanced the viral load in the fish liver, kidney, and spleen. The oxidative-antioxidant balance suggested that AFB1 induced severe oxidative stress, including increased reactive oxygen species (ROS) and malondialdehyde (MDA) levels in the AFB1 exposed group, and the reduced activity of superoxide dismutase (SOD), glutathione-S-transferase (GST) and catalase (CAT) in the AFB1 exposed group. Meanwhile, the related expression of nuclear factor erythroid 2-related factor 2 (Nrf2), interferon regulatory factor 3 (IRF3) and the type 1 interferon (IFN1) were noticeably down-regulated, but caspase-1 was up-regulated, after exposure to AFB1, demonstrating that fish are unable to avoid the virus infection. It should be noted that the intestinal microbiota diversity and richness were lower in the AFB1 exposed group, and the composition of intestinal microbiota was affected by AFB1, resulting in the higher abundance of bacteria (such as *Aeromonas* and *Bacteroides*) and the lower abundance of potentially beneficial bacteria (such as *Cetobacterium* and *Clostridium*) in the AFB1 exposed group. This research provides insight into the possibility that AFB1 may increase the susceptibility of *C. gibelio* to CyHV-2 infection, and thus amplify the viral outbreak to endanger ecological safety in aquatic environment.

## 1. Introduction

In recent years, the rapid growth of the aquaculture industry worldwide, without an accompanying increase in fishmeal, has stimulated an increased use of plant ingredients as a source of protein in commercial aquaculture feeds [1]. Consequently, the potential risk of mycotoxin contamination in fish has increased due to the high amount of mycotoxin contamination in plant sources [2]. Mycotoxin, such as aflatoxin, exposure in fish causes growth inhibition, bioaccumulation, immunosuppression, and increased susceptibility to opportunistic pathogens [3]. Although certain precautions are taken at the feed production stage, mycotoxin contamination during transport and storage may be difficult to avoid [4]. Therefore, it is crucial to study the toxicity mechanism of mycotoxin on fish and find effective strategies to alleviate the adverse effects caused by mycotoxin.

Aflatoxins (AFs) are highly toxic, carcinogenic, teratogenic, and mutagenic secondary metabolites secreted primarily by conidial fungi of the genus *Aspergillus*, specifically *Aspergillus flavus* and *Aspergillus parasiticus* [5]. Aflatoxin has five main analogs: aflatoxin B1, G1, M1, B2, and G2 (AFB1, AFG1, AFM1, AFB2, and AFG2) [6]. These compounds are serious contaminants of food, water, aquafeeds, and aquaculture systems, causing health hazards in humans and animals [7]. The International Agency for Research on Cancer (IARC) classifies AFB1 and AFM1 as the most toxic and carcinogenic among different types of AFs [8]. Several studies around the world have reported that AFB1 has been detected at a ratio of 60–70% in aquaculture feeds [9,10,11,12]. Previous studies have reported that AFB1 causes internal organ dysfunction, including induction of hepatotoxicity, teratogenicity, and immunosuppression in fish [13,14]. AFB1 exposure could activate oxidative stress and the endoplasmic reticulum stress pathway, inducing apoptosis and inflammation in northern snakehead (*Channa argus*) [15]. Hepatic and intestinal histopathological damages were found in common carp (*Cyprinus carpio*) fed with an aflatoxin-contaminated diet [16]. Dietary aflatoxin B1 could decrease growth performance and damage the structural integrity of immune organs in juvenile grass carp (*Ctenopharyngodon idella*) [4]. Thus, an aflatoxin-contaminated diet may reduce the growth performance of fish and render them more susceptible to opportunistic pathogens frequently found in aquaculture systems [17].

The balance of pro-oxidation and anti-oxidation in the internal environment plays a key role in maintaining the immunity and metabolism function. The previous study demonstrated that AFB1 induced excessive production of reactive oxygen species (ROS) and hydrogen peroxide (H_2_O_2_) in the liver, and caused oxidative stress to aggravate liver damage [18]. DNA virus-induced ROS accumulation in the host could trigger the negative change of cyclic GMP-AMP (cGAMP) synthase (cGAS) and nuclear factor erythroid 2-related factor 2 (Nrf2), which is associated with type-I interferon regulatory factor 3 (IRF3) activation, and ultimately affect the secretion of type I IFNs needed to achieve viral immune escape [19,20,21]. Therefore, the immune organ damage caused by AFB1 may be related to the reduction of the antioxidant capacity [4].

Intestinal bacteria play an important role in host health owing to the critical effect on metabolism and immune function [22]. Cumulative evidence demonstrates that the intestinal microbiota can modulate IFN responses, indirectly affecting viral infections [23,24,25]. External factors may change the structure of gut microbiota, which in turn affect intestinal physiological function [26]. Previous studies have reported that AFB1 could reduce the diversity of the composition of the intestinal microbiota community and severely affect the gut microbiota metabolome in rats [27]. However, research on the effects of aflatoxin on the intestinal flora in aquatic animals is lacking.

Gibel carp, *Carassius auratus gibelio*, is one of the most important cultivated freshwater fish species in China [28]. Crucian carp hematopoietic necrosis is an acute and contagious hemorrhagic disease in crucian carp caused by cyprinid herpesvirus 2 (CyHV-2) [29]. In recent years, CyHV-2 has spread rapidly in many provinces of China and resulted in huge losses for the crucian carp farming industry [30].

In the present study, we characterized the defensive capacities of gibel carp exposed to AFB1 and subsequently challenged them with CyHV-2 infection. To comprehensively evaluate the threat of AFB1 to aquaculture, the oxidative stress, gut microbiota, and related gene expression were assessed.

## 2. Materials and Methods

### 2.1. Fish Specimens

Healthy gibel carp specimens (15 ± 2 g, 12 ± 1 cm) with no history of disease were obtained from the gibel carp breeding base in Wuhan, China. The fish were maintained in recirculating aquaria (300 L) for 14 days to acclimatize to laboratory conditions. During the acclimatization period, the fish were fed with commercial feed (Tongwei, Chengdu, China) twice a day, the water temperature was 25 °C ± 1 °C and the water was renewed daily, approximately 30%. All animal experimental procedures were conducted according to the Animal Experimental Ethical Inspection (Ethical protocol code: YFI2022-zhouyong-09).

### 2.2. Diet Preparation and Sampling

AFB1 (purity > 98%) was purchased from Sigma–Aldrich (Sigma–Aldrich, St. Louis, MI, USA). The fish were randomly selected and divided into three groups: Group 1 (control diet, C), Group 2 (control diet + 50 µg/kg AFB1, T1), and Group 3 (control diet + 100 µg/kg AFB1, T2). There were three replicates in each group, with 60 fish in each replicate.

On days 14 and 28 of feeding, three fish from each tank were randomly collected and anesthetized with 100 mg/L MS222 (Sigma Aldrich, St. Louis, MI, USA). Over the 28-day period, excluding sampled fish, the survival rate of the control, T1, and T2 fish was 98.8%, 97.2%, and 95.6%, respectively. The liver tissues were removed and divided into three parts. One part was fixed in neutral 4% paraformaldehyde and subsequently used for immunofluorescence, one part was placed in RNase-free centrifuge tubes containing 200 µL TRIzol reagent (Invitrogen, Carlsbad, CA, USA) and subsequently used to analyze related genes expression, and the third part was placed in sterile tubes and stored at −80 °C to be used to determine the liver antioxidant index. During the 28 days of feeding, intestinal tissue was flash-frozen in liquid nitrogen and stored at −80 °C for Illumina sequencing.

### 2.3. Immunofluorescence

Liver tissues were fixed in 4% paraformaldehyde for 24 h and dehydrated in a sequential ethanol series. Tissue blocks were sectioned on a freezing microtome (Olympus, Tokyo, Japan). The tissue sections were blocked in 5% bovine serum albumin with normal serum in 0.1% Triton X-100, washed, and incubated with primary antibodies (CAT, 1:1000, ABCAM, Cambridge, MA, USA; GSTT1, 1:1000). The tissue sections were further incubated with secondary antibodies (Alexa Fluor 555-conjugated antibodies and Alexa Fluor 488-conjugated antibodies, 1:500, Invitrogen). Cell nuclei were stained with DAPI solution (blue). Images were obtained using fluorescence microscopy (Olympus BX41).

### 2.4. Changes in Antioxidant and Antiviral Related Genes Expression

The TRIzol reagent (Invitrogen, Carlsbad, CA, USA) was used to extract total RNA from tissues of gibel carp. The quality and purity of RNA were assessed by nanodrop (Thermo, Waltham, MA, USA). The cDNA was synthesized using random primers (TaKaRa, Dalian, China) following the manufacturer’s instructions. Then, the cDNA was used as the template for real-time quantitative PCR by real-time PCR Kit (TaKaRa, Dalian, China). The β-actin gene was performed as an internal reference. The specific primers are listed in Table 1. Each PCR reaction was performed in triplicate. The relative quantification of gene expression was conducted using the 2^−ΔΔCT^ method [31].

### 2.5. Liver Antioxidant Index

Liver tissues were homogenized in phosphate-buffered saline at a ratio of 1:9 (*w*/*v*) using a glass homogenizer at 4 °C. The homogenate was centrifuged at 5000× *g* for 20 min at 4 °C to remove tissue debris. The supernatant was used to determine the superoxide dismutase (SOD) activity, malondialdehyde (MDA) levels, and reactive oxygen species (ROS) levels using appropriate kits according to the manufacturer’s instructions (Jiancheng, Nanjing, China).

### 2.6. Illumina miSeq Sequencing and Bioinformatics Analysis

The total bacterial genomic DNA of the intestinal content was extracted using a Bacterial DNA Kit (Omega, Norcross, GA, USA) following the manufacturer’s instructions. The DNA was quantified using NanoDrop 2000 spectrometer (Thermo Fisher Scientific, Waltham, MA, USA), diluted to a concentration of 1 ng/µL, and stored at −20 °C. The V3–V4 region of the bacterial 16S rRNA gene was amplified by PCR using specific primers 338F (5′-ACTCCTACGGGAGGCAGCA-3′) and 806R (5′-GGACTACHVGGGTWTCTAAT-3′) with barcodes in 50 µL reactions. The following thermal cycling conditions were used: initial denaturation at 95 °C for 1 min; followed by 30 cycles at 95 °C for 30 s, 55 °C for 30 s, and 72 °C for 45 s; and a final extension at 72 °C for 10 min. PCR amplicons were isolated from 2% agarose gels and purified using the DNA Gel Extraction Kit (Omega, Norcross, USA). Subsequently, amplicons were sequenced on an Illumina MiSeq PE250 high-throughput sequencing platform. All sequence reads were quality filtered and assembled using the Mothur software package [39]. Reads were clustered into operational taxonomic units (OTUs) at 97% identity [40]. Representative reads were selected from each OTU using QIIME package. Annotated taxonomic classification was performed using the RDP Classifier algorithm (http://rdp.cme.msu.edu/ accessed on 20 August 2022) against the Silva database version 123 (16S rDNA) [41].

Alpha diversity analyses (abundance-based coverage estimator [ACE], Chao, Shannon, and Simpson index) were performed using Mothur (version v.1.30) [42]. Beta diversity was estimated by computing the Bray-Curtis distance based on the abundances of microbes at the genus level and visualized using principal coordinate analyses (PCoA) and Unweighted Pair Group Method with Arithmetic Mean (UPGMA) clustering. Other analyses were performed using the R software.

### 2.7. Challenge Tests and Viral Load in Liver, Kidney, and Spleen of Gibel Carp

After 28 days of the feeding trial, a challenge test was performed in each group with CyHV-2 purified by sucrose gradient ultracentrifugation in our laboratory. Fish in each group were intraperitoneally injected with 0.2 mL CyHV-2 (10^6.3^ TCID_50_/mL). Infected fish were observed daily, and mortality was recorded for 14 days. At 2, 7, and 14 days post injection (dpi), three fish were randomly selected to investigate the viral load. Liver, kidney, and spleen tissues were collected from the fish. Total DNA was extracted from each tissue type using a Viral DNA Kit (Omega, Norcross, GA, USA). Viral DNA was quantified using droplet digital PCR (ddPCR, Bio-Rad, Hercules, CA, USA) with the primers CyHV2-F, CyHV2-R, and CyHV2-probe (Table 1) [29]. The viral load was determined as the number of viral copies per microgram of total tissue.

### 2.8. Statistical Analysis

Data were analyzed by one-way analysis of variance (ANOVA) and expressed as the arithmetic mean ± standard deviation (SD). Survival curves were estimated by the Kaplan–Meier method. Differences were determined by Tukey’s test in SPSS statistical software (SPSS Inc., Chicago, IL, USA), with *p*-values < 0.05 indicating statistical significance.

## 3. Results

### 3.1. Oxidative Stress in the Liver

**Figure 1:**
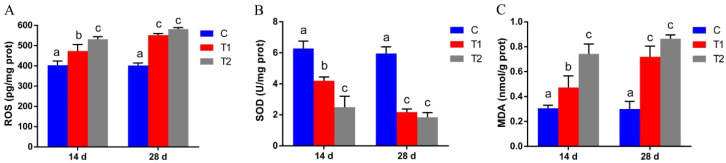
Reactive oxygen species (ROS; (**A**)), superoxide dismutase (SOD; (**B**)), and malondialdehyde (MDA; (**C**)) activity in gibel carp liver after exposure to aflatoxin B1 (AFB1) for 14 and 28 days. C: control diet, T1: 50 µg/kg AFB1, T2: 100 µg/kg AFB1. Results are presented as mean ± standard deviation (SD). Dissimilar superscript letters represent statistically significant differences between different treatment groups (*p* < 0.05).

**Figure 2:**
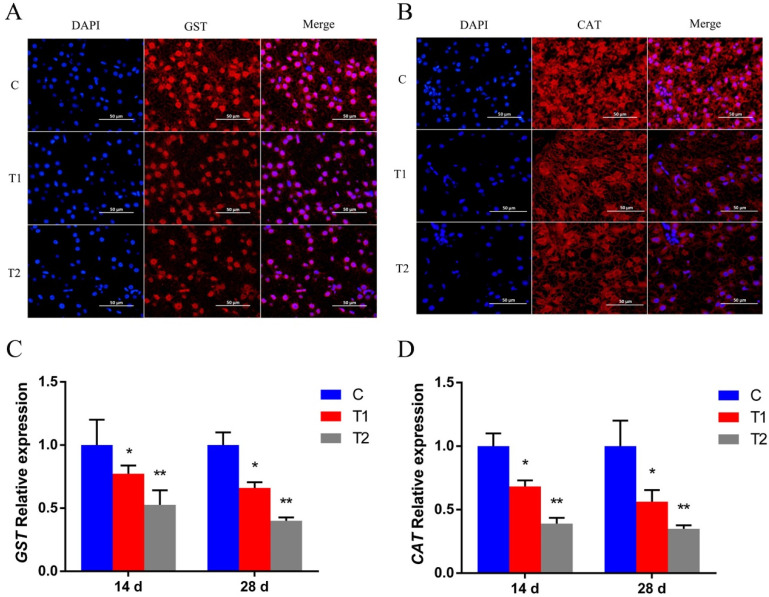
Effects of aflatoxin B1 (AFB1) on glutathione-S-transferase (GST) and catalase (CAT) levels in the liver of gibel carp. (**A**,**B**) Immunofluorescence of GST and CAT in the liver of gibel carp. (**C**,**D**) mRNA expression levels of the *GST* and *CAT* in the liver of gibel carp. C: control diet, T1: 50 µg/kg AFB1, T2: 100 µg/kg AFB1.Data are presented as mean ± standard deviation (SD; * *p* < 0.05; ** *p* < 0.01).

As shown in Figure 1, the 50 μg/kg AFB1 and 100 μg/kg AFB1 exposed groups showed significantly higher ROS and MDA levels and significantly lower SOD levels in the liver than those in the control group (C; *p* < 0.05; Figure 2A–C). At day 28, the ROS and MDA levels in the 100 μg/kg AFB1 exposed group were about 45% and 189% higher, respectively, and the mean SOD levels in the 100 μg/kg AFB1-exposed group were 45% of the control group. There were no significant differences between the 50 μg/kg AFB1 and 100 μg/kg AFB1 exposed groups on day 28 (*p* > 0.05).

### 3.2. Effects of Aflatoxin B1 on Antioxidant Enzymes in the Liver

Liver tissue obtained from the AFB1 exposed groups on day 28 showed a lower intensity of red fluorescence (Figure 2A,B) than the control group, indicating lower GST and CAT levels in hepatocytes. Moreover, the fluorescence intensity was lower in 100 μg/kg AFB1 exposed group than in 50 μg/kg AFB1 group. The GST and CAT mRNA expression levels in the liver were significantly lower in the 50 μg/kg AFB1 and 100 μg/kg AFB1 exposed groups than in the control group on days 14 and 28 (*p* < 0.05; Figure 2C,D).

### 3.3. Effects of Aflatoxin B1 on Related Genes Expression

**Figure 3:**
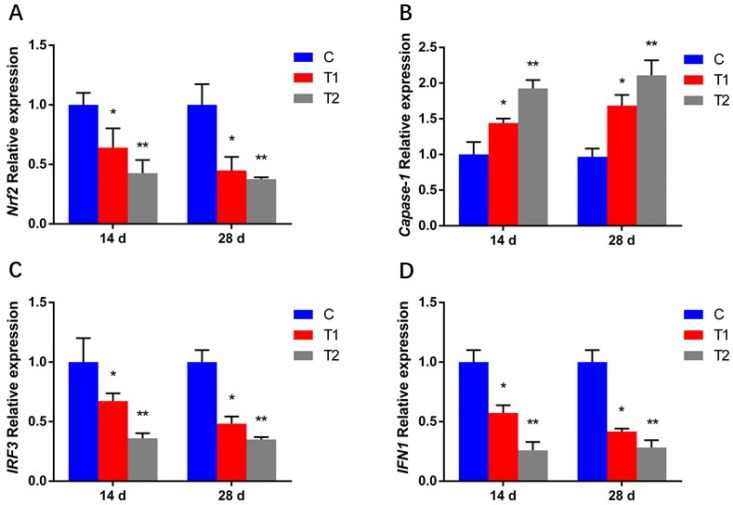
Effects of AFB1 on the mRNA levels of Nrf2(**A**), Caspase-1(**B**), IRF3(**C**), and IFN1(**D**) in gibel carp. C: control diet, T1: 50 µg/kg AFB1, T2: 100 µg/kg AFB1. Data represent the means ± SD (* *p* < 0.05, ** *p* < 0.01).

The mRNA expressions of Nrf2, IRF3, and IFN1 were significantly lower in the AFB1 exposed groups on day 28 (*p* < 0.01) (Figure 3A,C,D), and at day 28, the mRNA expressions of apoptosis related gene (Caspase-1) were significantly higher in the AFB1 treatment groups than in the control group (*p* < 0.01) (Figure 3B).

### 3.4. Characteristics of 16S rDNA Sequencing

**Figure 4:**
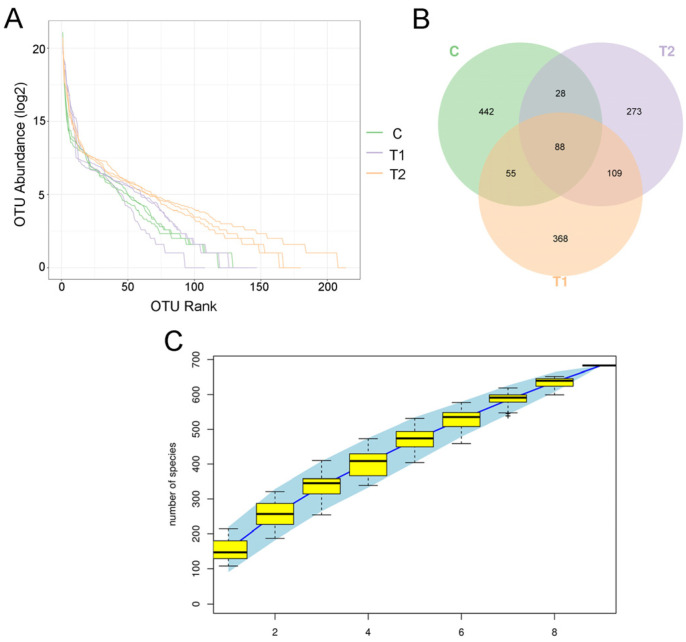
Rarefaction curves, Venn diagram, and rank abundance curve of different treatment groups of gibel carp exposed to aflatoxin B1 for 28 days. (**A**) Rarefaction curves. (**B**) Venn diagram representing the operational taxonomic units (OTUs) shared among treatment groups. (**C**) Rank abundance curve. C: control diet, T1: 50 µg/kg AFB1, T2: 100 µg/kg AFB1.

After quality filtering and assignment, 990,671 valid read sequences were obtained from the nine samples belonging to three groups. The rarefaction curves and rank abundance curve demonstrated that sufficient sequencing depth, richness, and evenness were achieved for each sample (Figure 4A,C).

On the basis of 97% nucleotide sequence identity, these high-quality sequences were clustered into 1363 OTUs in total. The OTU distribution in different groups is depicted in a Venn diagram in Figure 4B. The number of OTUs in the control group was higher than that in the treatment groups. Meanwhile, 88 core OTUs were observed in all collected samples. Furthermore, the number of OTUs in the 100 μg/kg AFB1 exposed group was the lowest.

### 3.5. Effects of Aflatoxin B1 on Alpha Diversity of Intestinal Microbiota

**Figure 5:**
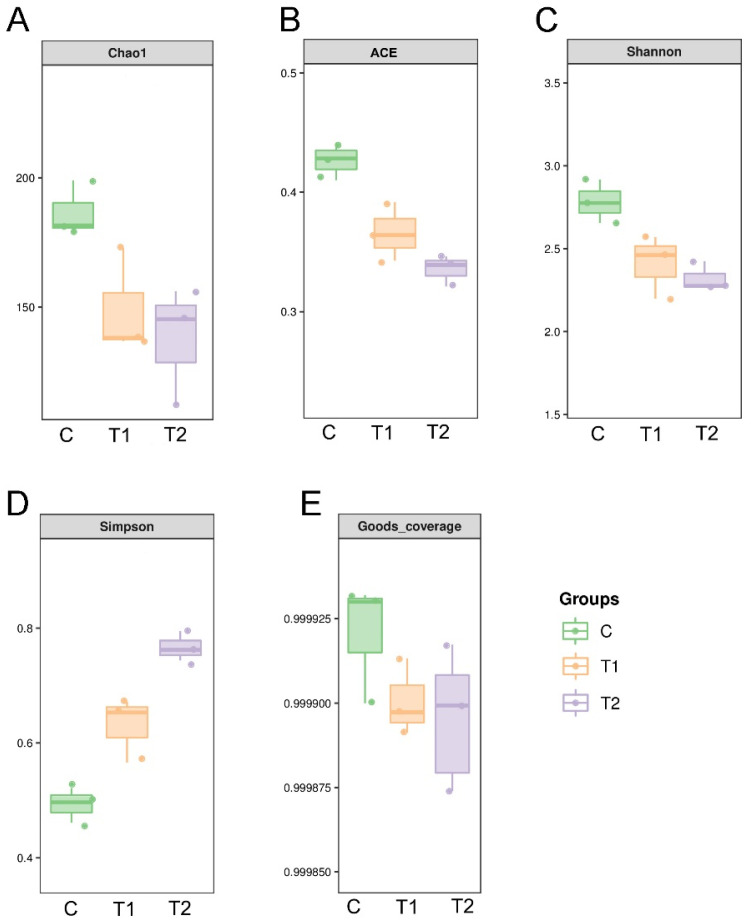
Richness and diversity indices of bacterial communities in different treatment groups of gibel carp exposed to aflatoxin B1 for 28 days. (**A**) Chao1 index, (**B**) abundance-based coverage estimator (ACE), (**C**) Shannon index, and (**D**) Simpson index of operational taxonomic unit (OTU) level. (**E**) The average Good’s coverage of treatment groups. C: control diet, T1: 50 µg/kg AFB1, T2: 100 µg/kg AFB1.

ACE and Chao1 indices were used to quantify species richness. The ACE index ranged from 0.34 to 0.44, and the Chao1 index ranged from 37 to 198. The ACE index and Chao1 index were significantly lower in the AFB1 exposed groups than in the control group (C; *p* < 0.05; Figure 5A,B). The alpha diversity of each sample was calculated via the Shannon index and Simpson index. The Shannon index ranged from 2.2 to 2.8, and the Simpson index ranged from 0.43 to 0.79. The AFB1 exposed groups showed a significantly lower Shannon index and a significantly higher Simpson index than the control group (Figure 5C,D). The richness and diversity of bacterial communities in the treatment groups were lower than those in control group. The average Good’s coverage was 0.999913 (values ranged from 0.999870 to 0.99936), indicating that the sequences identified represented the majority of the bacteria in each sample (Figure 5E).

### 3.6. Microbial Community Composition

**Figure 6:**
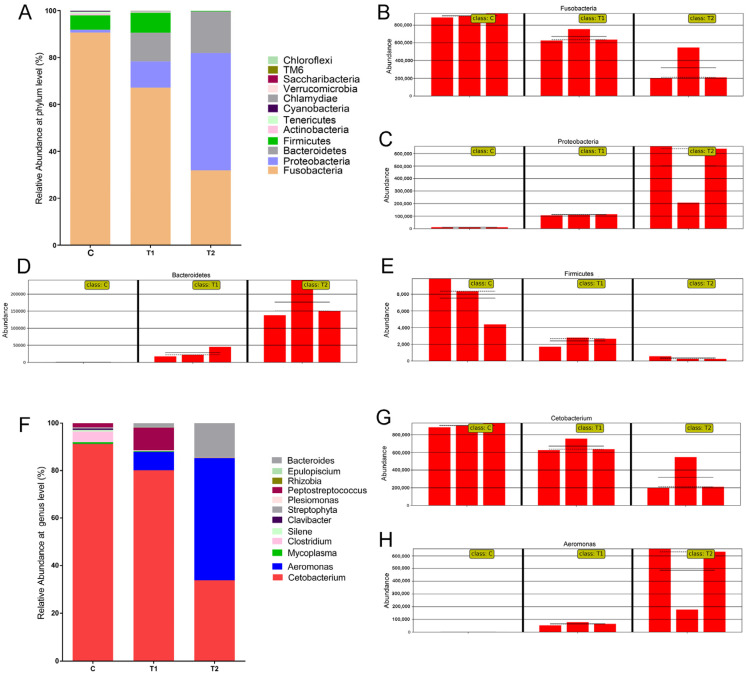
Gut microbiota composition at the phylum level (**A**) and at the genus level (**F**) in gibel carp exposed to aflatoxin B1 for 28 days. (**B**–**E**) Partial phyla with significant changes in relative abundance among treatment groups. (**G**,**H**) Partial genera with significant changes in relative abundance among treatment groups. C: control diet, T1: 50 µg/kg AFB1, T2: 100 µg/kg AFB1.

A total of 19 phyla were identified in all samples, and five phyla were identified at an abundance >1%. As shown in Figure 6A, the dominant phyla in the control group were Fusobacteria, Proteobacteria, Firmicutes, and Actinobacteria, accounting for over 98% of the bacterial sequences from control group samples. In the treatment groups, the dominant bacterial phyla were Fusobacteria, Proteobacteria, Bacteroidetes, and Firmicutes, accounting for over 99% of the total reads from treatment group. At the phylum level, the AFB1 exposed groups showed a significantly higher abundance of Proteobacteria and Bacteroidetes (*p* < 0.05; Figure 6C,D) and a significantly lower abundance of Fusobacteria and Firmicutes (*p* < 0.05; Figure 6B,E) than the control group.

A total of 198 genera were detected across all nine samples. In the control group, the dominant genera were *Cetobacterium*, *Clostridium*, *Peptostreptococcus*, and *Mycoplasma* (Figure 6F). In the treatment groups, the dominant genera were *Cetobacterium*, *Aeromonas*, *Bacteroides*, and *Peptostreptococcus*. The relative abundance of dominant genera of intestinal bacteria was significantly different between the control and the treatment groups. At the genus level, the AFB1 exposed groups showed a significantly higher abundance of *Aeromonas* (*p* < 0.05; Figure 6C,D), and a significantly lower abundance of *Cetobacterium* (*p* < 0.05; Figure 6B,E) than the control group.

### 3.7. Beta Diversity of Intestinal Microbiota

**Figure 7:**
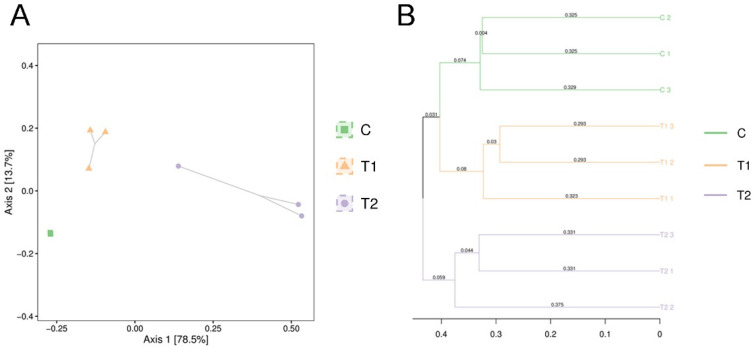
Beta diversity of the gut microbiota among different groups of gibel carp exposed to aflatoxin B1. (**A**) The principal co-ordinate analysis (PCoA) of the bacterial community at the operational taxonomic unit (OTU) level. (**B**) The hierarchical clustering tree was calculated using the unweighted pair group method with arithmetic mean (UPGMA) method. C: control diet, T1: 50 µg/kg AFB1, T2: 100 µg/kg AFB1.

PCoA and unweighted pair group method with arithmetic mean (UPGMA) clustering were conducted to evaluate beta diversity. As shown in Figure 7, the intestinal microbiomes of the control group and treatment group were separated into different clusters. In addition, the individual differences in gut microflora in the AFB1 exposed groups were less than those in the control group. Overall, the PCoA and UPGMA clustering results showed that AFB1 exposure markedly altered the intestinal microbial community structure in gibel carp.

### 3.8. Effect of AFB1 on CyHV-2 Infection and Viral Load in Tissues in Gibel Carp

**Figure 8:**
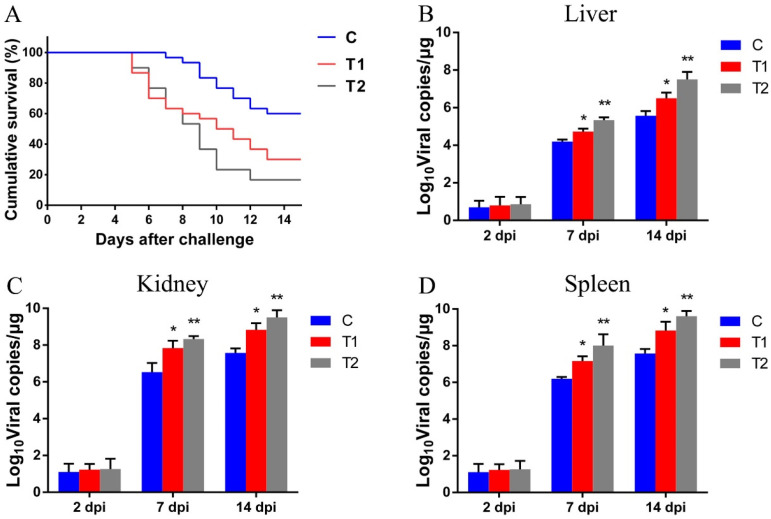
Effects of AFB1 on cyprinid herpesvirus 2 (CyHV-2) infection in gibel carp and viral load in tissues of gibel carp. (**A**) Cumulative survivorship curves of gibel carp intraperitoneally injected with CyHV-2 in different treatment groups. (**B**–**D**) CyHV-2 viral load in the liver, kidney, and spleen of gibel carp in different treatment groups at 2, 7, and 14 days post infection (dpi). C: control diet, T1: 50 µg/kg AFB1, T2: 100 µg/kg AFB1. Data are presented as mean ± standard deviation (SD; * *p* < 0.05; ** *p* < 0.01).

The cumulative survival rate of *C. gibelio* challenged with CyHV-2 for 14 days is shown in Figure 8A. The cumulative mortality of CyHV-2-infected gibel carp in the control group was 40%, and the cumulative mortality in the T2 group reached 83% at 14 dpi. At 14 dpi, the cumulative survival rate of fish exposed to AFB1 was significantly less than in the control group (*p* < 0.05). Moreover, the 100 μg/kg AFB1 exposed group of gibel carp showed significantly higher mortality than the 50 μg/kg AFB1 exposed group. Regarding viral load, the AFB1 exposed groups showed a significantly higher number of copies of CyHV-2 in the liver, kidney, and spleen than the control group (*p* < 0.05; Figure 8B–D).

## 4. Discussion

Fisheries provide a large amount of high-quality protein to meet the nutritional requirements of the increasing human population (Food and Agriculture Organization [FAO], Rome, Italy, 2018). The rapid expansion of fish farming has led to an increased use of plant protein sources in aquaculture feeds [13]. AFB1 could be a latent threat to the health of aquatic organisms when fishmeal ingredients are replaced with plant-based materials in aquafeeds [43]. The present study reveals that AFB1 causes oxidative stress, changes in intestinal microbiota and exacerbates CyHV-2 infection in *C. gibelio*.

Oxidative stress under exposure to extreme conditions induces ROS and MDA production, which may lead to protein, lipid, and DNA damage [44]. In the present study, changes in the levels of antioxidant enzymes and oxidative stress markers were observed in the AFB1 exposed groups. The lower SOD levels, and the higher ROS and MDA levels were observed in the AFB1 exposed groups. The immunofluorescence and mRNA expression levels of GST and CAT showed that the quantity of antioxidant enzymes was lower in the AFB1 exposed groups. These results suggest that AFB1 induces oxidative stress. Moreover, the oxidative stress increased with the increase in AFB1 concentration. Similarly, previous studies have reported that AFB1 significantly reduced the activity of antioxidant enzymes by down-regulating the expression of Nrf2, and increased the levels of ROS and MDA in Nile tilapia [45] and gibel carp [46]. Previous research showed that ROS accumulation could promote the replication of CyHV-2, while antioxidants could inhibit the amplification of CyHV-2 by activating Nrf2 signaling pathway [19]. In this study, we found that the expression of Nrf2 was inhibited significantly by AFB1. Thus, we believe that the oxidative stress induced by AFB1 could promote CyHV-2 proliferation.

**Figure 9:**
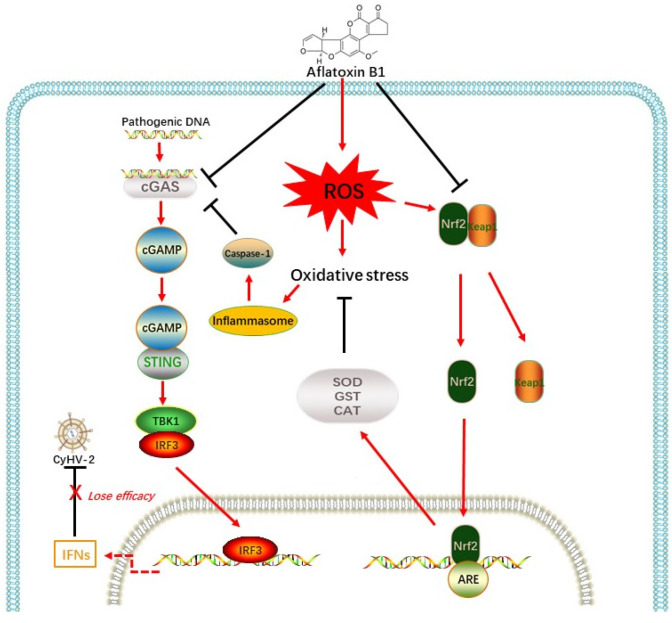
Proposed model for AFB1 inducing the oxidative stress and promoting CyHV-2 proliferation through suppressing the IFNs response [19,20,66].

Recently, it has been reported that ROS accumulation could limit the virus DNA induced cGAS-STING activation by activating caspase-1 [20]. STING is involved in the activation of IRF3. Activated IRF3 induces inflammation through generating IFN1 [21]. Our study demonstrated that the expression of caspase-1 was higher, and the expressions of IRF3 and IFN1 were lower, in the AFB1 exposed group. Based on the challenge test, we found that AFB1 exposure increased the mortality of gibelio carp infected with CyHV-2 and enhanced the viral load in liver, kidney and spleen of gibelio carp. We believe that AFB1 promoted the amplification of CyHV-2 by inducing the oxidative stress and suppressing the IFN1 response (Figure 9). Similarly, it has been found that azoxystrobin enhance the spring viremia of carp virus (SVCV) replication by regulated the MAPK-Nrf2 signaling pathway to suppress HO-1-mediated IFN expression [47].

Intestinal microbiota is crucial to host health owing to its significant influence on metabolism and immune function [48]. The gut microbiome can influence ROS levels, immune response and host health [49,50,51]. Changes in the intestinal flora are highly correlated with physiological, pathological, and environmental conditions [52]. An increasing number of studies have focused on the relationship between environmental pollution and gut microbiota to understand the toxicological response [53]. Bioaccumulation of benzophenone-3 in *Carassius auratus* can affect the structure and diversity of intestinal flora [54]. Another study showed that the intestinal microbiota of freshwater crayfish (*Procambarus clarkii*) was significantly altered by microcystin [55]. In the present study, the results demonstrated that AFB1 exposure disrupts the intestinal microbiota of gibel carp. The PCoA and UPGMA clustering analysis showed that AFB1 exposure markedly altered the intestinal microbial community structure of gibel carp. The AFB1 exposed groups showed significantly lower richness and diversity of the gut microbiota than the control group. The lower intestinal bacterial richness and diversity reduced the stability of intestinal microbial communities of gibel carp. Previous research suggests that viral infection, toxin exposure, and unfavorable environment reduce the richness and diversity of gut flora in animals [48,56,57]. In the present study, the intestinal microbiota composition of gibel carp was examined and compared to identify common flora that showed significant difference after AFB1 exposure. At the phylum level, the higher relative abundances of Proteobacteria and Bacteroidetes and the lower relative abundances of Fusobacteria and Firmicutes were observed in AFB1 exposed group. Some members of Proteobacteria and Bacteroidetes are opportunistic pathogens and facilitate inflammation or disrupt the intestinal mucosal barrier [58]. Fan et al. (2019) showed that increased relative abundance of phylum Proteobacteria in the intestine is associated with slow growth and disease in shrimp [59]. Previous studies have demonstrated that members of Fusobacteria regulate the transepithelial transport, thus strengthening the mucosal barrier and improving the oxidative and inflammatory status of the intestinal mucosa [60]. At the genus level, the abundance of *Aeromonas* was significantly higher and that of *Cetobacterium* was significantly lower in the AFB1 exposed groups than in the control group. Members of *Aeromonas* are ubiquitous opportunistic pathogens in the intestinal tracts of aquatic animals and aquaculture waters, and some of these species cause infections in humans [61]. *Cetobacterium* has been observed to be the dominant genus in the intestinal microbiota of different freshwater fishes; it has been shown to improve digestion and produce large quantities of vitamin B [62]. Several studies pointed to the role of the intestinal microbiota in modulating the systemic immunity and providing a competitive barrier to bacterial, viral, and fungal pathogens [23,63]. In murine models of lymphocytic choriomeningitis virus (LCMV) or influenza infection, disorder of the gut microbiota results in an unresponsive reaction to the virus [64]. Feeding with *Clostridium butyricum* improved the host immune responses and the survival rate of gibel carp against *Carassius auratus* herpesvirus (CaHV) infection [65]. Our data demonstrated that the changes in intestinal microbiota caused by AFB1 might weaken the immunity and antioxidant capacity of gibel carp.

## 5. Conclusions

In summary, our study is the first, to our knowledge, to report that AFB1 exacerbates viral infection in aquatic animals. We found that AFB1 exposure increased the mortality and enhanced the viral load of gibelio carp infected with CyHV-2. The cCAS-STING pathway and the expression of IFN1 was significantly suppressed by AFB1, which might be associated with severe oxidative stress and intestinal microbiota disorder induced by AFB1. This research provides new understanding of the threat that AFB1 may increase CyHV-2 susceptibility in *C. gibelio*.

## Figures and Tables

**Table 1:** Primer sequences used in this study.

Gene	Primer Sequence (5′-3′)	Accession Numbers	References
β-actin	F: CATCTACGAGGGTTACGCCC	NC068418.1	[32]
	R: AACCACACGTCGGCTTGTTA		
GST	F: CCTGAAAACAAACCGGCACA	NC068386.1	[32]
	R: AAAAGGAGGTGGCTCAACACG		
CAT	F: ATC TTACAGGAAACAACACCC	NC056596.1	[33]
	R: CGATTCAGGACGCAAACT		
Nrf2	F: GCGAGCGTAGCTCCAGTCTGA	MG759384.1	[34]
	R: AAGGCTTGCCGTGCTCGTCT		
IRF3	F: TCCAGGCCAAGCATACGAA	NC056583.1	[35]
	R: CCATTTGCAACAGCCATCAT		
Caspase-1	F: AAACCCAAGATCATCATCATCCA	NW024042261.1	[36]
	R: CAGGGCATCAGCCTCTAAGTTGT		
IFN1	F: GTCAATGCTCTGCTTGCGAAT	NC007114.7	[37]
	R: CAAGAAACTTCACCTGGTCCT		
CyHV-2	F: TCGGTTGGACTCGGTTTGTG		
	R: CTCGGTCTTGATGCGTTTCTTG	AY939863.1	[38]
CyHV-2 probe	FAM-CCGCTTCCAGTCTGGGCCACTACC-BHQ1		

## Data Availability

The data presented in this study are available on request from the corresponding author.
